# The germline mutational landscape of *BRCA1* and *BRCA*2 in Brazil

**DOI:** 10.1038/s41598-018-27315-2

**Published:** 2018-06-15

**Authors:** Edenir Inêz Palmero, Dirce Maria Carraro, Barbara Alemar, Miguel Angelo Martins Moreira, Ândrea Ribeiro-dos-Santos, Kiyoko Abe-Sandes, Henrique Campos Reis Galvão, Rui Manuel Reis, Cristiano de Pádua Souza, Natalia Campacci, Maria Isabel Achatz, Rafael Canfield Brianese, Maria Nirvana da Cruz Formiga, Fabiana Baroni Makdissi, Fernando Regla Vargas, Anna Cláudia Evangelista dos Santos, Hector N. Seuanez, Kelly Rose Lobo de Souza, Cristina B. O. Netto, Patrícia Santos-Silva, Gustavo Stumpf da Silva, Rommel M. R. Burbano, Sidney Santos, Paulo Pimentel Assumpção, Izabel Maria Monteiro Bernardes, Taisa Manuela Bonfim Machado-Lopes, Thais Ferreira Bomfim, Maria Betânia Pereira Toralles, Ivana Nascimento, Bernardo Garicochea, Sergio D. Simon, Simone Noronha, Fernanda Teresa de Lima, Anisse Marques Chami, Camila Matzenbacher Bittar, Jose Bines, Osvaldo Artigalas, Maria Del Pilar Esteves-Diz, Tirzah Braz Petta Lajus, Ana Carolina Leite Vieira Costa Gifoni, Rodrigo S. C. Guindalini, Terezinha Sarquis Cintra, Ida V. D. Schwartz, Pricila Bernardi, Diego Miguel, Sonia Tereza dos Santos Nogueira, Josef Herzog, Jeffrey N. Weitzel, Patricia Ashton-Prolla

**Affiliations:** 1 Molecular Oncology Research Center, Barretos Cancer Hospital, Barretos, Brazil; 2 Barretos School of Health Science, Faculdade de Ciências da Saúde de Barretos Dr. Paulo Prata, Barretos, Brazil; 3 International Research Center/CIPE, AC Camargo Cancer Center, Sao Paulo, Brazil; 4 Programa de Pós-Graduação em Genética e Biologia Molecular, Universidade Federal do Rio Grande do Sul, Porto Alegre, Brazil; 5 Laboratório de Medicina Genômica, Hospital de Clínicas de Porto Alegre, Porto Alegre, Brazil; 6 Programa de Genética, Instituto Nacional de Câncer, Rio de Janeiro, Brazil; 7 Laboratório de Genética Humana e Médica, Programa de Pós-graduação em Genética e Biologia Molecular - Universidade Federal do Pará, Belém do Pará, Brazil; 8 Núcleo de Pesquisas em Oncologia, Programa de Pós-graduação em Genética e Biologia Molecular - Universidade Federal do Pará, Belém do Pará, Brazil; 9 Laboratório de Imunologia e Biologia Molecular, Universidade Federal da Bahia, Salvador, Brazil; 10 Department of Oncogenetics, Barretos Cancer Hospital, Barretos, Brazil; 11 Life and Health Sciences Research Institute (ICVS), Health Sciences School, University of Minho, Braga, Portugal; 12 ICVS/3B’s-PT Government Associate Laboratory, Braga, Guimarães Portugal; 13 Clinical Genetics Branch, Division of Cancer Epidemiology and Genetics - Department of Health and Human Services/Centro de Oncologia, NCI-NIH/Hospital Sírio Libanês, Bethesda, USA; 14 Oncogenetics and Clinical Oncology Departments, AC Camargo Cancer Center, São Paulo, Brazil; 15 Breast Surgery Department, AC Camargo Cancer Center, São Paulo, Brazil; 16 Birth Defects Epidemiology Laboratory, Instituto Oswaldo Cruz, Fundação Oswaldo Cruz, Rio de Janeiro, Brazil; 17 Genetics and Molecular Biology Department, Universidade Federal do Estado do Rio de Janeiro, Rio de Janeiro, Brazil; 18 Serviço de Genética Médica, Hospital de Clínicas de Porto Alegre, Porto Alegre, Brazil; 19 Laboratório de Biologia Molecular, Hospital Ophir Loyola, Belem do Pará, Brazil; 20 Núcleo de Oncologia da Bahia - Grupo Oncoclínicas, Salvador, Brazil; 21 Centro de Paulista de Oncologia, Oncoclínicas, São Paulo, Brazil; 22 Departamento de Oncologia Clínica, Hospital Israelita Albert Einstein, São Paulo, Brazil; 23 COAEM - Centro Oncológico Antonio Ermirio de Moraes, São Paulo, Brazil; 24 Centro de Aconselhamento Genético, Hospital Israelita Albert Einstein, São Paulo, Brazil; 25 Rede Mater Dei de Saúde, Belo Horizonte, Brazil; 26 Instituto Hermes Pardini, Belo Horizonte, Brazil; 27 Oncopraxis, Rio de Janeiro, Brazil; 28 Hospital Moinhos de Vento (HMV), Porto Alegre, Brazil; 29 Departamento de Radiologia e Oncologia, Instituto do Câncer do Estado de São Paulo/Faculdade de Medicina da Universidade de São Paulo, São Paulo, Brazil; 30 Departamento de Biologia Celular e Genética - Serviço de Aconselhamento Genético, Centro de Oncologia Avançado/CECAN, Universidade Federal do Rio Grande do Norte - Hospital Liga Contra o Câncer, Natal, Brazil; 31 Rede D’Or (Fujiday and OncoStar), Fortaleza, Brazil; 32 Oncocentro, Hospital São Carlos, Fortaleza, Brazil; 33 CLION, CAM Group, Salvador, Brazil; 34 Laboratório Genoma, Vitória, Brazil; 35 Serviço de Genética Médica do Hospital Universitário, Divisão de Clínica Médica, Universidade Federal de Santa Catarina, Florianópolis, Brazil; 36 Hospital Universitário Professor Edgard Santos, Serviço de Genética Médica, Universidade Federal da Bahia, Salvador, Brazil; 37 Departamento de Oncogenética, Oncoclin de Manaus, Manaus, Brazil; 38 Department of Population Sciences, Division of Clinical Cancer Genomics - City of Hope, Duarte, USA

## Abstract

The detection of germline mutations in *BRCA1* and BRCA*2* is essential to the formulation of clinical management strategies, and in Brazil, there is limited access to these services, mainly due to the costs/availability of genetic testing. Aiming at the identification of recurrent mutations that could be included in a low-cost mutation panel, used as a first screening approach, we compiled the testing reports of 649 probands with pathogenic/likely pathogenic variants referred to 28 public and private health care centers distributed across 11 Brazilian States. Overall, 126 and 103 distinct mutations were identified in *BRCA1* and *BRCA2*, respectively. Twenty-six novel variants were reported from both genes, and *BRCA2* showed higher mutational heterogeneity. Some recurrent mutations were reported exclusively in certain geographic regions, suggesting a founder effect. Our findings confirm that there is significant molecular heterogeneity in these genes among Brazilian carriers, while also suggesting that this heterogeneity precludes the use of screening protocols that include recurrent mutation testing only. This is the first study to show that profiles of recurrent mutations may be unique to different Brazilian regions. These data should be explored in larger regional cohorts to determine if screening with a panel of recurrent mutations would be effective.

## Introduction

*BRCA1* and *BRCA2* are tumor suppressor genes and their protein products play an important role in the repair of DNA double-strand breaks through homologous recombination (HR)^1^. Individuals harboring germline pathogenic variants in *BRCA1* and *BRCA2* (*BRCA*) are strongly predisposed to the development of breast (BC; lifetime risk up to 85% and 45%, respectively) and ovarian cancers (OC; lifetime risk up to 39% and 11%, respectively)^2^ as well as other solid tumors^3^. As *bona fide* tumor-suppressor genes, the wild-type allele is frequently lost (mainly through loss of heterozygosity) during tumorigenesis, thereby becoming completely inactivated in the tumor^4^. Tumors arising within the context of a complete loss of *BRCA* function are amenable to treatment with agents targeting HR DNA repair deficiency, such as poly(ADP) ribose polymerase (PARP) inhibitors (PARPi) and platinum-based chemotherapy^5^. In the past few years, these drugs have shown to be effective in the treatment of advanced ovarian cancer in patients harboring somatic and/or germline *BRCA* mutations^6,7^, and more recently, the FDA extended the approval of PARPi in the BC *BRCA*-related metastatic clinical setting^8,9^. These approvals will benefit many patients since *BRCA* mutations are identified in 8–13% of all ovarian cancer cases^10^, and approximately 10% of breast cancers^11^, which is the most common cancer type among women worldwide and also in Brazil^12,13^.

Considering this scenario, the identification of a *BRCA* mutation is of paramount importance not only for providing appropriate genetic counseling and discussing risk-reducing interventions, but also for determining treatment options in patients with metastatic disease. A challenge in the identification of carriers in Latin America, however, is the limited availability of cancer risk evaluation programs and genetic testing *per se*. In Brazil, 70–80% of the population relies on the public health care system^14^, which does not provide genetic testing. Consequently, the mutational profile of *BRCA* remains largely unknown. Systematic testing of at-risk individuals brings knowledge of the genetic background of a population, and may enable the identification of multiple recurrent and/or founder mutations, which, in turn, would support the use of mutation panels as a first-line screening tool.

In this study, we aimed to describe the landscape of *BRCA* germline mutations in Brazil and investigate if the use of a panel of recurrent mutations would be useful in this population.

## Results

**Figure 1:**
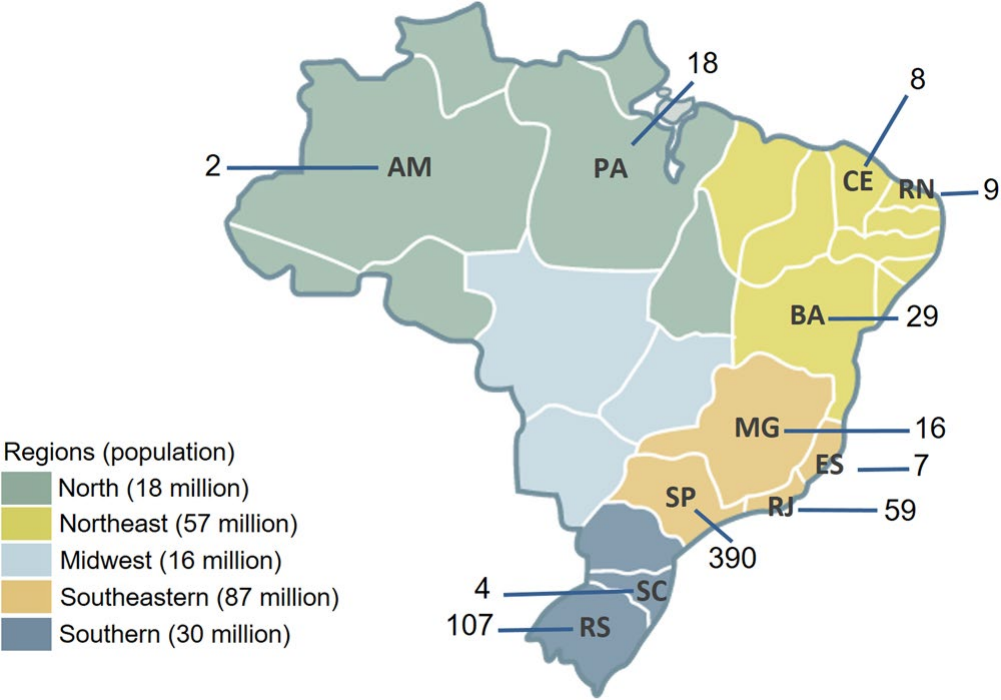
Geographical distribution of HBOC patients with pathogenic and likely pathogenic *BRCA1* and *BRCA2* variants in Brazil (N = 649). Legends represent the Brazilian States of Amazonas (AM), Pará (PA), Ceará (CE), Rio Grande do Norte (RN), Bahia (BA), Minas Gerais (MG), Espírito Santo (ES), Rio de Janeiro (RJ), São Paulo (SP), Santa Catarina (SC) and Rio Grande do Sul (RS) and the numbers indicate the number of cases reported from each State. The five Brazilian regions are depicted in different colors and the number between parentheses indicate the approximated population of each region.

A total of 649 reports of pathogenic/likely pathogenic variants were retrieved from 28 centers in 11 different Brazilian States. As shown in Fig. 1, the majority of reports was obtained from the State of São Paulo (60.1%), which is also the State with the largest number of participating centers (N = 7). The second largest number of reports was obtained from the State of Rio Grande do Sul (16.5%).

**Figure 2:**
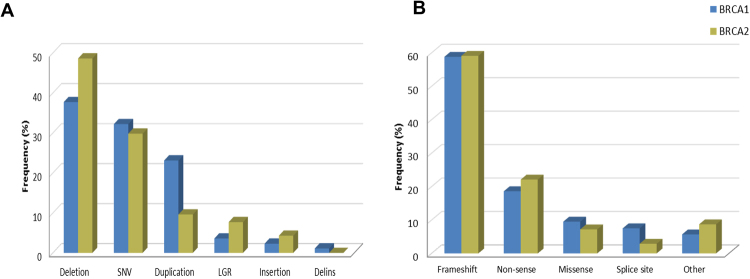
Frequency of each type of variation and molecular consequence among reported *BRCA1* and *BRCA2* mutations. SNV, single nucleotide variants; LGR, large genomic rearrangements.

The most common types of pathogenic variants identified in both genes were small deletions and single nucleotide variants (SNVs), and are predicted to result in frameshift and non-sense alterations in the protein sequence (Fig. 2). Synonymous pathogenic variants were identified only once in each gene, in two distinct patients: *BRCA1* c.4185G>A and the *BRCA2* c.9117G>A. Large genomic rearrangements (LGR) were present in 4.9% of all cases, and among them, the *BRCA2* c.156_157insAlu corresponded to 34.3% of all LGRs. The frequency of each type of variant and their molecular consequences are depicted in Fig. 2A,B, respectively.

**Figure 3:**
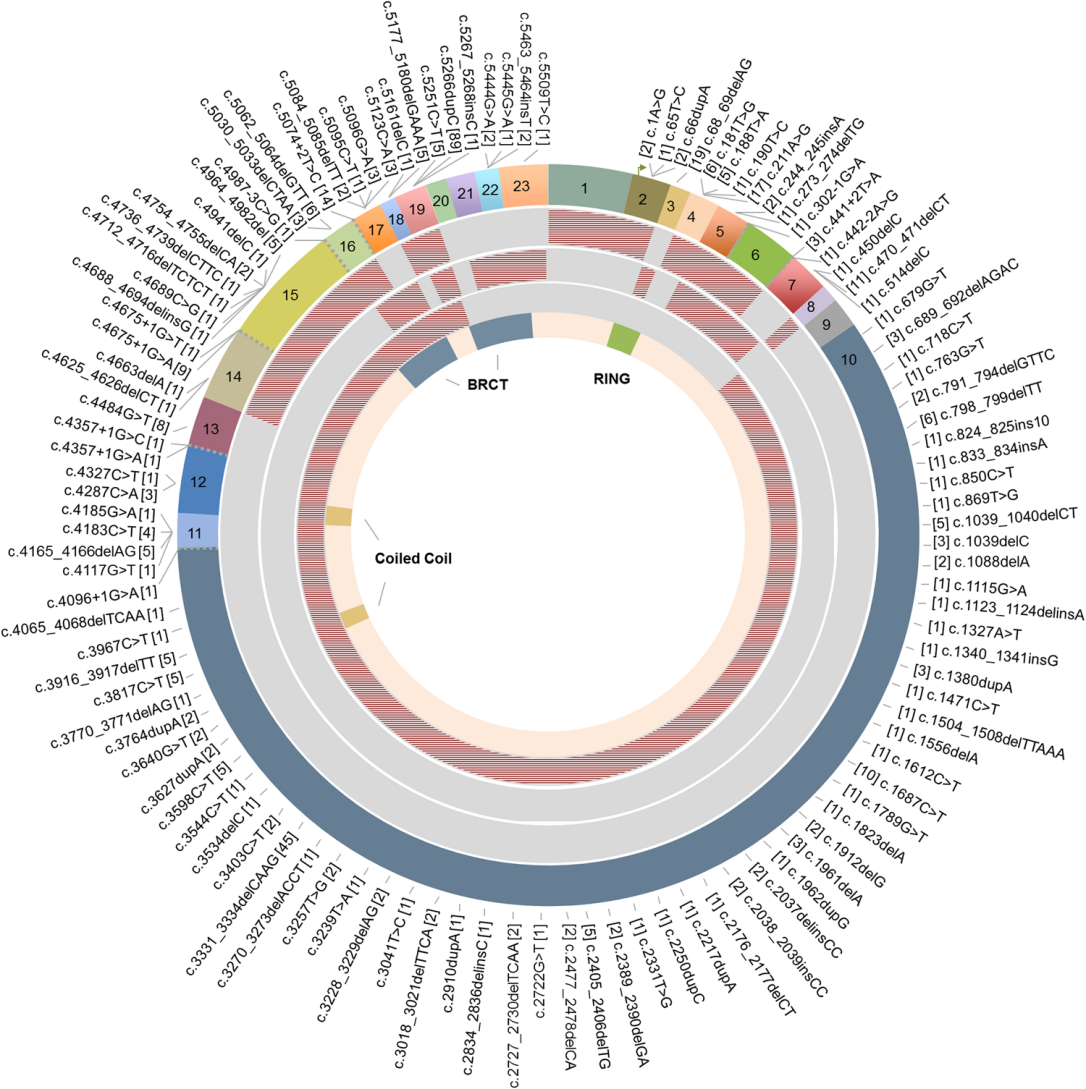
Circos plot showing the distribution of all reported *BRCA1* mutations. Point mutations and small deletions and insertions are shown around in the outermost ring, which represents the *BRCA1* exons. The number between brackets correspond to the number of mutation carriers. Each reported LGR is represented by dashed blocks in the three intermediate rings, while the innermost ring represent the *BRCA1* domains.

**Figure 4:**
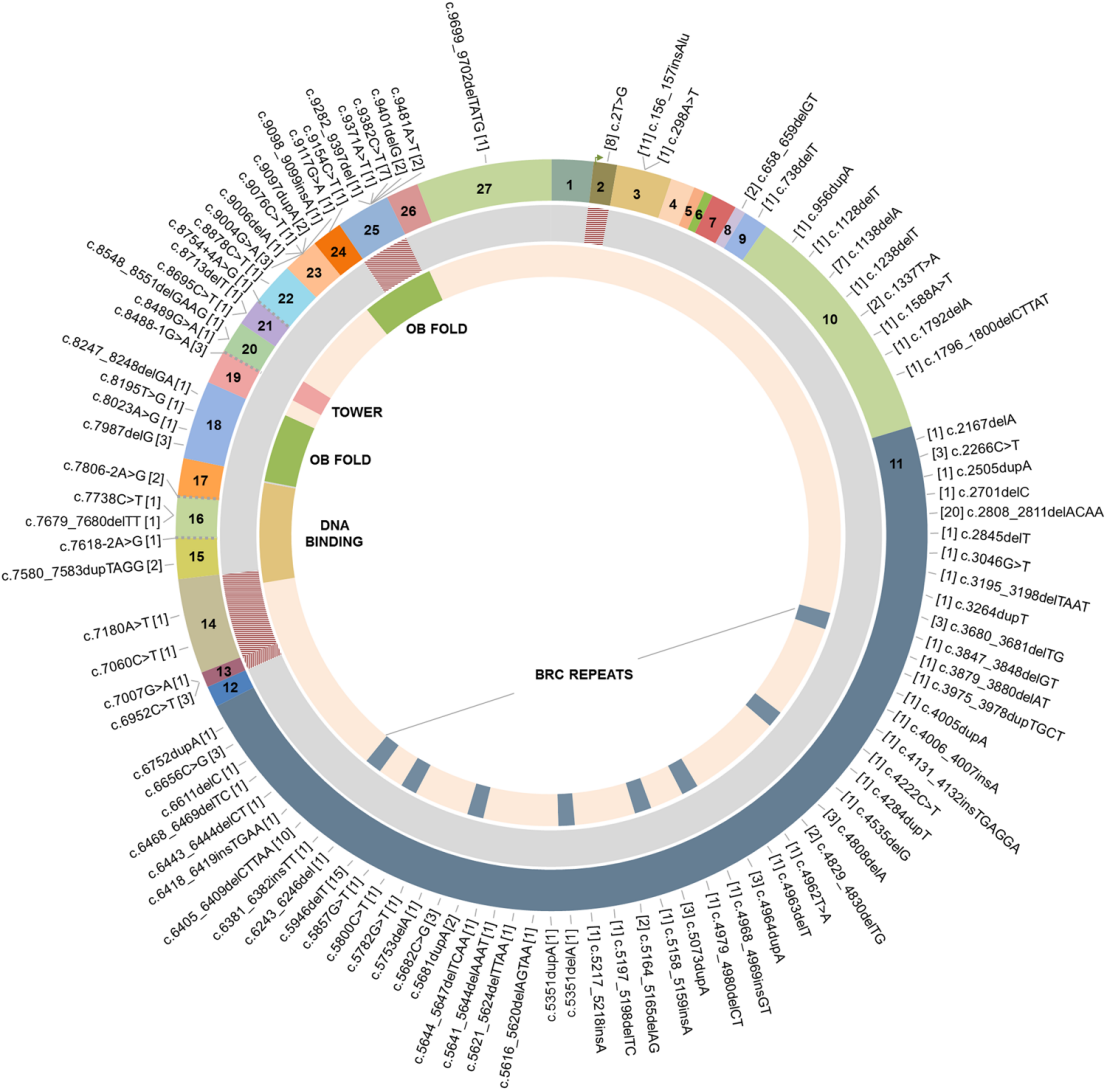
Circos plot showing the distribution of all reported *BRCA2* mutations. Point mutations and small deletions and insertions are shown around in the outermost ring, which represents the *BRCA2* exons. The number between brackets correspond to the number of mutation carriers. Each reported LGR is represented by dashed blocks in the intermediate ring, while the innermost ring represent the *BRCA2* domains.

As shown in Table 1, 126 distinct pathogenic *BRCA1* variants were identified among 441 probands, corresponding to 68% (441/649) of all reported mutations. Among these, a subset of 33 distinct *BRCA1* mutations corresponded to 73.4% of all variants identified in this gene. The nine most prevalent *BRCA1* mutations accounted for 50.3% of all *BRCA1* reported mutations, and among these, the European founder mutation c.5266dupC (formerly known as 5382insC) was the most common, corresponding to 20.2% of all variants found in *BRCA1*. In *BRCA2*, 103 distinct variants were identified in 208 probands, corresponding to 32% of all individuals tested (Table 2). The mutational profile of *BRCA2* was more heterogeneous, since non-recurring mutations (those seen only once) were more common (35.1%) than in *BRCA1* (15.4%). Moreover, a higher frequency of novel variants was identified in *BRCA2* (17/103) when compared to *BRCA1* (9/126). Figures 3 and 4 show all reported *BRCA1* and *BRCA2* mutations, respectively, including LGR in both genes. Detailed information about *BRCA1* and *BRCA2* mutations (predicted protein change, rs number and overall frequency) are summarized in the Supplementary Dataset.

**Table 1:** Reported mutations in *BRCA1*, showing 126 distinct mutations identified in 441 unrelated individuals.

Mutations identified in one proband (N = 68; 15.4%)		Mutations identified in two probands (N = 25; 11.3%)	Mutations identified in three or more probands (N = 33; 73.4%), N and (%)		
c.65T>C	c.3534delC	c.1A>G	c.5266dupC	89	(20.2)
c.190T>C	c.3544C>T	c.66dupA	c.3331_3334delCAAG	45	(10.2)
c.273_274delTG	c.3627dupA	**c.244_245insA**	c.68_69delAG	19	(4.3)
c.302-1G>A	c.3770_3771delAG	c.791_794delGTTC	c.211A>G	17	(3.9)
**c.442-2A>G**	c.3967C>T	c.1088delA	c.5074 + 2T>C	14	(3.2)
c.450delC	c.4065_4068delTCAA	c.1912delG	c.470_471delCT	11	(2.5)
c.514delC	c.4096 + 1G>A	c.2037delinsCC	c.1687C>T	10	(2.3)
c.679G>T	c.4117G>T	c.2038_2039insCC	c.4675+1G>A	9	(2.0)
c.718C>T	c.4185G>A	c.2389_2390delGA	c.4484G>T	8	(1.8)
c.763G>T	c.4327C>T	c.2477_2478delCA	c.181T>G	6	(1.4)
c.824_825ins10	c.4357 + 1G>A	c.2727_2730delTCAA	c.798_799delTT	6	(1.4)
c.833_834insA	c.4357 + 1G>C	c.3018_3021delTTCA	c.5062_5064delGTT	6	(1.4)
c.850C>T	c.4625_4626delCT	c.3228_3229delAG	c.188T>A	5	(1.1)
c.869T>G	**c.4663delA**	c.3257T>G	c.1039_1040delCT	5	(1.1)
c.1115G>A	c.4675 + 1G>T	c.3403C>T	c.2405_2406delTG	5	(1.1)
**c.1123_1124delinsA**	c.4688_4694delinsG	c.3640G>T	c.3598C>T	5	(1.1)
c.1327A>T	c.4689C>G	c.3627dupA	c.3817C>T	5	(1.1)
c.1340_1341insG	c.4712_4716delTCTCT	c.3764dupA	c.3916_3917delTT	5	(1.1)
c.1471C>T	**c.4736_4739delCTTC**	c.4754_4755delCA	c.4165_4166delAG	5	(1.1)
c.1504_1508delTTAAA	c.4941delC	c.5084_5085delTT	c.4964_4982del	5	(1.1)
c.1556delA	c.4987-3C>G	c.5444G>A	c.5177_5180delGAAA	5	(1.1)
c.1612C>T	c.5095C>T	c.5463_5464insT	c.5251C>T	5	(1.1)
c.1789G>T	**c.5161delC**	Deletion exon 1–2	c.4183C>T	4	(0.9)
c.1823delA	c.5267_5268insC	Deletion exon 5–7	c.689_692delAGAC	3	(0.7)
c.1962dupG	c.5445G>A	Deletion exon 21–23	c.441+2T>A	3	(0.7)
c.2176_2177delCT	c.5509T>C		c.1039delC	3	(0.7)
c.2217dupA	Deletion exon 3		c.1380dupA	3	(0.7)
**c.2250dupC**	Deletion exon 4–6		c.1961delA	3	(0.7)
c.2331T>G	Deletion exon 8		c.4287C>A	3	(0.7)
c.2722G>T	Deletion exon 9–19		c.5030_5033delCTAA	3	(0.7)
c.2834_2836delinsC	Deletion exon 14–16		c.5096G>A	3	(0.7)
**c.2910dupA**	Deletion exon 16–17		c.5123C>A	3	(0.7)
c.3041T>C	Deletion exon 18–19		Deletion exon 19	3	(0.7)
c.3239T>A					
**c.3270_3273delACCT**					

Mutations in bold are novel (not described in ClinVar, BRCA Share, LOVD, ARUP or BRCA Exchange database) and underlined mutations were described in other database but not in ClinVar. Frequencies and proportions (%) in each column correspond to the fraction of each group among all *BRCA1* variants identified (N = 441). See Supplementary Dataset for detailed information.

**Table 2:** Reported mutations in *BRCA2*, showing 103 distinct mutations identified in 208 unrelated individuals.

Mutations identified in one proband (N = 73; 35.1%)		Mutations identified in two probands (N = 12; 11.5%)	Mutations identified in three or more probands (N = 18; 53.4%), N and (%)		
c.298A>T	**c.5753delA**	c.658_659delGT	c.2808_2811delACAA	20	(9.6)
c.738delT	c.5782G>T	**c.1337T>A**	c.5946delT	15	(7.2)
c.956dupA	c.5800C>T	c.4829_4830delTG	c.156_157insAlu	11	(5.3)
c.1128delT	c.5857G>T	c.5164_5165delAG	c.6405_6409delCTTAA	10	(4.8)
c.1238delT	**c.6243_6246del**	c.5681dupA	c.2T>G	8	(3.8)
c.1588A>T	**c.6381_6382insTT**	**c.7580_7583dupTAGG**	c.1138delA	7	(3.4)
c.1792delA	**c.6418_6419insTGAA**	c.7806-2A>G	c.9382C>T	7	(3.4)
c.1796_1800delCTTAT	c.6443_6444delCT	c.9097dupA	c.2266C>T	3	(1.4)
**c.2167delA**	c.6468_6469delTC	c.9098_9099insA	c.3680_3681delTG	3	(1.4)
**c.2505dupA**	c.6611delC	c.9401delG	c.4808delA	3	(1.4)
c.2701delC	**c.6752dupA**	c.9481A>T	c.4964dupA	3	(1.4)
c.2845delT	c.7007G>A	Deletion exon 2	c.5073dupA	3	(1.4)
c.3046G>T	c.7060C>T		c.5682C>G	3	(1.4)
c.3195_3198delTAAT	c.7180A>T		c.6656C>G	3	(1.4)
c.3264dupT	c.7618-2A>G		c.6952C>T	3	(1.4)
c.3847_3848delGT	c.7679_7680delTT		c.7987delG	3	(1.4)
**c.3879_3880delAT**	c.7738C>T		c.8488-1G>A	3	(1.4)
c.3975_3978dupTGCT	c.8023A>G		c.9004G>A	3	(1.4)
c.4005dupA	c.8195T>G				
c.4006_4007insA	c.8247_8248delGA				
c.4131_4132insTGAGGA	c.8489G>A				
c.4222C>T	c.8548_8551delGAAG				
c.4284dupT	c.8695C>T				
c.4535delG	**c.8713delT**				
c.4962T>A	c.8754+4A>G				
c.4963delT	c.8878C>T				
**c.4968_4969insGT**	**c.9006delA**				
**c.4979_4980delCT**	c.9076C>T				
**c.5158_5159insA**	c.9117G>A				
c.5197_5198delTC	c.9154C>T				
**c.5217_5218insA**	**c.9282_9397del**				
c.5351delA	c.9371 A > T				
c.5351dupA	c.9699_9702delTATG				
c.5616_5620delAGTAA	Deletion exon 13				
c.5621_5624delTTAA	Deletion exon 14				
c.5641_5644delAAAT	Deletion exon 25				
c.5644_5647delTCAA					

Mutations in bold are novel (not described in ClinVar, BRCA Share, LOVD, ARUP or BRCA Exchange database). Frequencies and proportions (%) in each column correspond to the fraction of each group among all *BRCA2* variants identified (N = 208). See Supplementary Dataset for detailed information.

Although the most common mutation, *BRCA1* c.5266dupC, was reported in all geographical regions, some recurrent *BRCA1* mutations (detected in three or more individuals) seem to be unique to a particular Brazilian State. The variants c.188 T>A, c.2405_2406delTG, c.3916_3917delTT, c.689_692delAGAC, c.4287C>A, and c.5123C>A were reported exclusively among individuals recruited from the State of São Paulo (Southeastern region). In addition, the c.1039_1040delCT and c.1039delC variants were reported exclusively in the State of Pará (Northern region), while c.3598C>T and c.5177_5180delGAAA were only reported in pathogenic mutation carriers from the State of Rio Grande do Sul (Southern Region). No similar trends were observed among *BRCA2* recurrent mutations.

When considering all *BRCA1* and *BRCA2* mutations seen in three or more individuals, a subset of 51 variants (33 in *BRCA1* and 18 in *BRCA2)*, accounted for 67% of all reports. In a more stringent scenario, mutations seen in four or more individuals, totaling 30 variants (23 in *BRCA1* and 7 in *BRCA2*) corresponded to 57.3% of all mutations.

## Discussion

Many factors affect the probability of developing breast or ovarian cancer, but no predictor is as determinant and prevalent as the inheritance of a *BRCA* mutation. There are several clinical management options for individuals harboring *BRCA* mutations, including risk reducing surgeries (bilateral risk-reducing mastectomy, salpingo-oophorectomy)^15^, chemoprevention^16^ and intensive surveillance with annual breast magnetic resonance imaging^17^. Several studies have demonstrated that, after identifying a *BRCA*-mutation carrier, genetic counseling and testing of at-risk individuals results in increased surveillance and use of risk-reduction strategies ultimately leading to primary or secondary prevention of cancer and improved outcomes in carriers^18^. Despite these benefits there is limited availability of genetic testing in Latin American countries, including Brazil^14,19^.

Low cost screening panels including recurrent *BRCA* pathogenic variants (e.g. Ashkenazi Jewish Panel) have been used in certain countries/populations as an initial approach to overcome technical and economical restrictions that still exist for comprehensive *BRCA1* and *BRCA2* testing. Most of the populations where this strategy is used show few mutations occurring at a high frequency, often due to founder effects^19,20^. Thus, the development of such panels depends on a deep knowledge of the mutational spectrum of the target population and the presence of a relatively small number of recurrent mutations explaining a significant proportion of cases. This strategy has been proposed, for instance, for Hispanic breast and/or ovarian cancer families (with predominantly Mexican origin) where nine recurrent variants account for 53% of all detected *BRCA* mutations^21^. For this population, a low-cost multiplex PCR-based panel (HISPANEL) was developed and subsequently estimated to identify up to 75% of all true Mexican *BRCA* mutations. The pattern of highly recurrent mutations is also seen in other Latin American countries: Bahamas (six recurrent mutations correspond to 89.4% of all carriers), Colombia (three recurrent mutations correspond to 88.9% of all carriers) and Peru (three recurrent mutations correspond to 84.6% of all carriers)^19^. However, this striking pattern of recurrent mutations seen in several Latin American countries may not be observed in all countries, and specific mutations maybe be shared only by a few populations. In fact, a recent Brazilian study showed that the use of a single screening panel for different Latin American populations will likely not be effective^22^, because there does not seem to be a significant overlap of recurrent mutations among different Latin American populations^19,23^. These results are not surprising due to vary distinct population migration waves and therefore genetic admixture background of Brazil in comparison with the other Latin American countries^24^.

Knowledge about the germline mutational spectrum among Brazilian HBOC patients is limited. Only five studies have performed comprehensive *BRCA* mutation testing (using gene sequencing and LGR analysis) to date^25–29^, corresponding to only 1,041 individuals tested, among a Brazilian population of over 207 million people^30^. Most studies have focused on specific mutations, or screened only a few regions of *BRCA1* and/or *BRCA2* (summarized in the Supplementary Dataset). To our knowledge, this is the largest comprehensive description of the spectrum of germline *BRCA* mutations in different geographical Brazilian regions.

Most of the mutations reported previously in smaller Brazilian studies, involving the analysis of only certain gene regions, have also been identified in our cohort, but it is noteworthy that some of the previously reported recurrent mutations are completely absent in this dataset. The most striking example is the *BRCA1* ins6Kb rearrangement, which was reported by Esteves *et al*.^31^ in seven carriers, five of whom were from Rio Grande do Sul State. However, in our cohort we did not identify this rearrangement in any patient, even considering that Rio Grande do Sul was the second State in terms of the number of reported carriers (N = 107). The *BRCA1* 6 kb insertion can be detected by routine LGR testing through MLPA, and although we cannot assure that all probands were subjected to MLPA analysis, we can expect that most of them with negative sequencing results were also investigated for LGRs, since patients from the private healthcare setting and also those enrolled in research studies are routinely tested for LGR by MLPA. Indeed, a recent study from Alemar *et al*. reported LGR data from 351 HBOC probands from the same Brazilian State where the previous cases harboring the 6 kb insertion were reported originally, and the *BRCA1* 6 kb insertion was not detected, suggesting a very low frequency of this LGR in probands with the HBOC phenotype^29^.

Among all distinct mutations identified 11.8% were novel, corresponding to 4.6% of all carriers and highlighting the heterogeneity of our population. The identification of novel mutations linked to HBOC is a vital information that should be shared with established mutation databases, in order to become useful for interpreting further tests and to answer questions about the association between a variant and phenotype.

Overall our data show a significant molecular heterogeneity among the *BRCA1* and *BRCA2* mutations identified, and a similar profile of type and molecular consequence of pathogenic variants in both genes. In addition, *BRCA1* mutations were more frequent than *BRCA2* mutations, which is in agreement with previous data showing this same proportion of mutations between both genes among women from different ethnicities, except Asians^32^. Also, similar to previous report^33^, the rate of large genomic rearrangements did not exceed 5% of all mutations. However, it is remarkable that 34.3% of all LGR reported here correspond to the Portuguese founder mutation *BRCA2* c.156_157insAlu^34^. Although significant, this frequency may still be an underestimation, since until very recently the detection of this particular mutation, which requires a specific PCR reaction, was not carried out by most commercial laboratories. Recently (July 2016), MRC-Holland included an extra probe that detects the wild-type sequence of this region in its *BRCA2* MLPA kits (P090 version B1 and P45 version C1), allowing the detection of this variant during MLPA testing. This simple modification is expected to increase significantly the detection rate of c.156_157insAlu in Brazilian HBOC patients. The probands with c.156_157insAlu identified here were from the States of Minas Gerais (1), Rio de Janeiro (3) Rio Grande do Sul (2) and São Paulo (5) but screening for this LGR should be done for patients regardless of State of origin.

In the current HBOC genetic testing landscape, where most laboratories have migrated to next generation sequencing (NGS), analysis workflows allow the filtering of many types of mutations, including the exclusion of synonymous variants. In our study, we have identified two pathogenic synonymous mutations, and this finding highlights the importance of careful evaluation of each *BRCA* variant detected. It is widely known that synonymous substitutions can alter splicing accuracy, creating or destroying a native donor or acceptor splice site, but they can also can modify translation fidelity, mRNA structure and protein folding^35^. Indeed, both pathogenic synonymous variants identified in this study disrupt splice donor sites, leading to exon skipping. The G nucleotide of *BRCA1* c.4185G>A represents the last nucleotide of exon 11 (according to LRG nomenclature, formerly known as exon 12), and is conserved in 86% of the splice sites in mammals^36^. This variant lead to an aberrant transcript lacking exon 12^37^. Similarly, the *BRCA2* c.9117G>A leads to a complete deletion of exon 23^38^ and produces a frameshift effect similar to other deleterious mutations^39^. The process of evaluating variant significance should include multiple databases, as four mutations reported here were not described in ClinVar, although classified as clearly pathogenic in other databases. Finally, even variants described in one database should have their significance confirmed in other databases, especially if classified as variants of uncertain significance (VUS). As an example, using an *ex vivo* assay based on a splicing reporter minigene, Brandão *et al*.^40^ demonstrated that the *BRCA1* c.4987-3C>G variant leads to the skipping of exon 17. However, it remains classified as VUS in ClinVar and it is not reported in other databases.

In this study, we have attempted to compile pathogenic and likely pathogenic *BRCA1* and *BRCA2* variants identified in the main Genetic Cancer Risk Assessment centers in Brazil. Although this report in fact is the most comprehensive to date, both in number of mutations reported, as well as in number of centers/regions of the country included, many limitations must be considered when analyzing the results. We were unable to obtain information on the birthplace for most of the carriers, which would have been more informative than the center where the genetic test was performed. Therefore, data on geographical location should be interpreted with caution. In fact, among some of the individuals tested in the State of São Paulo we were able to identify residents from the Midwest States. This is not unexpected since the paucity of clinical and laboratory personnel trained in clinical cancer genetics in Brazil, results in a pattern of patients with suspected hereditary cancer being refered to testing from different parts of the country to only a few reference centers very distant from their residence place. Moreover, it might be possible that the inclusion of data from point mutation analysis could increase the detection and, consequently, the reporting of a few specific mutations. However, our data regarding the most frequently reported mutations is in agreement with previous Brazilian studies that performed full *BRCA1* and *BRCA2* sequencing and MLPA. These studies show, for example, that the *BRCA1* c.5266dupC is the most prevalent mutation across distinct regions of Brazil, which was also the case in our study^26,27,29^.

We confirm that there is significant molecular heterogeneity in the *BRCA1* and *BRCA2* genes among Brazilian carriers. Although our findings suggest that this heterogeneity precludes the use of screening protocols that include recurrent mutation testing only, our results also show that certain mutations occur at a high frequency in some Brazilian regions and not others. These variations could be due to mutation founder effects, which have been described for other genes in Brazil. These findings should be explored in larger cohorts from specific Brazilian regions to assess whether in these areas, screening with a panel of recurrent mutations would be effective.

## Materials and Methods

Laboratory reports of *BRCA1* and *BRCA2* testing showing pathogenic or likely pathogenic germline mutations were compiled from 28 public and private health care offices located in 11 Brazilian states, including the main reference centers for Genetic Cancer Risk Assessment (GCRA) in Brazil. Not all probands were subjected to a comprehensive *BRCA* testing (full *BRCA* sequencing and multiplex ligation-dependent probe amplification, MLPA). The genetic testing was performed using distinct methodologies, including full gene analysis by Sanger or next generation sequencing, point mutation analysis by Sanger or genotyping methods (as HISPANEL), and MLPA for analysis of large genomic rearrangements. Most data came from institutions participating in the Brazilian Hereditary Cancer Network (BHCN), convened by the Brazilian National Cancer Institute (INCA, Instituto Nacional de Cancer) and partially supported by public funding from the National Council for Scientific and Technical Development (CNPq)^41^. These centers, mostly public hospitals, are established in the Cities/States of Belém/Pará (in the Northern region, encompassing the Amazon basin), Salvador/Bahia (in the Northeastern region), Vitória/Espírito Santo, Rio de Janeiro/Rio de Janeiro, São Paulo/São Paulo, Ribeirão Preto/São Paulo, Barretos/São Paulo (in the Southeastern region) and Porto Alegre/Rio Grande do Sul (in Southern Brazil). In addition, public or private health care offices from the States of Amazonas (Northern region and the Amazon basin), Minas Gerais (Southeastern region), Rio Grande do Norte, Ceará (Northeastern region) and Santa Catarina (Southern region) also contributed with molecular data from their patients (Fig. 1). All subjects were unrelated and fulfilled HBOC criteria for *BRCA* testing. Some of the mutations described in this manuscript were also described in prior population/region-specific prevalence studies^22,25,26,33,42–45^. This project was approved by the Institutional Review Board from Hospital de Clínicas de Porto Alegre (approval n° 10-0521) and all individuals provided written or verbal consent for *BRCA* testing. All methods were performed in accordance with the relevant guidelines and regulations, and all data supporting the results are shown in the Supplementary Dataset.

The Human Genome Variation Society (HGVS) nomenclature guidelines (http://varnomen.hgvs.org/) were used to annotate identified variants and the ClinVar database (www.ncbi.nlm.nih.gov/clinvar/) was used to determine the biological significance of all reported variants. For novel variants, BRCA Share (formerly known as UMD, http://www.umd.be/), LOVD (http://www.lovd.nl/3.0/home), ARUP (http://arup.utah.edu/database/BRCA/) and BRCA Exchange (http://brcaexchange.org/) databases were also checked. Current ACMG^46^ guidelines were also used for further classification. BRCA1 and BRCA2 domains were defined using the boundaries in the Pfam database (http://pfam.xfam.org).

## Electronic supplementary material

Supplementary dataset 1
